# The power of ten: report from the 10th American Society for Microbiology Conference on Biofilms

**DOI:** 10.1128/jb.00235-26

**Published:** 2026-07-14

**Authors:** Caitlin Light, Megan O’Malley, Kendra Rumbaugh, Mette Burmølle, Birthe V. Kjellerup, Christophe Beloin, Clay Fuqua, Karin Sauer

**Affiliations:** 1 First-year Research Immersion Program, Binghamton Biofilm Research Center, Binghamton University 14787, Binghamton, New York, USA; 2 Department of Microbiology, University of Washington 312771, Seattle, Washington, USA; 3 Department of Surgery, Texas Tech University Health Sciences Center 740350, Lubbock, Texas, USA; 4 Department of Biology, Faculty of Science, University of Copenhagen 56603, Copenhagen, Denmark; 5 Department of Civil and Environmental Engineering, Clark School of Engineering, University of Maryland 688811 , College Park, Maryland, USA; 6 Genetics of Biofilms Laboratory, Institut Pasteur, Université de Paris Cité, UMR CNRS 6047 555089, Paris, France; 7 Department of Biology, Indiana University 123993, Bloomington, Indiana, USA; 8 Department of Biological Sciences, Binghamton Biofilm Research Center, Binghamton University 171431, Binghamton, New York, USA; Dartmouth College Geisel School of Medicine, Hanover, New Hampshire, USA

**Keywords:** biofilms, conference review

## Abstract

The 10th American Society for Microbiology Conference on Biofilms was held in person on 9–13 November 2025 in Portland, OR. The diverse mixture of over 300 oral and poster presentations resoundingly embodied the vitality of biofilm research across a wide range of topics and multiple scientific disciplines. Special activities, including a pre-conference symposium for early-career researchers, Lunch and Learn sessions, the Paul Stoodley imaging workshop, and various networking events, further enhanced the attendee experience. As a general theme, the conference was deliberately structured to provide high levels of participation and engagement among early-career scientists.

## INTRODUCTION

Biofilm research has entered a phase defined by mechanistic resolution and translational urgency. Presentations at the 10th ASM conference on Biofilms emphasized biofilms as highly structured, metabolically stratified communities whose matrix architecture, nutrient gradients, and stress-response networks drive their structure and antimicrobial tolerance. Biofilm research features more and more interdisciplinary approaches, ranging from microfluidics, spatial-omics, and advanced imaging to elucidate metabolic cross-feeding and niche-specific gene expression in biofilm communities. Current directions furthermore focused on pathways and emergent behaviors to identify actionable vulnerabilities and strategies to modulate biofilm behavior and resistance to therapeutic agents. Overall, it was clear that the field is converging on a systems-level understanding of biofilm physiology while accelerating the development of therapeutics and models that better reflect real-world infections. This review attempts to capture some of the tremendous energy and enthusiasm the conference generated and describes some of the new and exciting science that was presented.

## CONFERENCE STRUCTURE AND TOPICS COVERED

**Fig 1:**
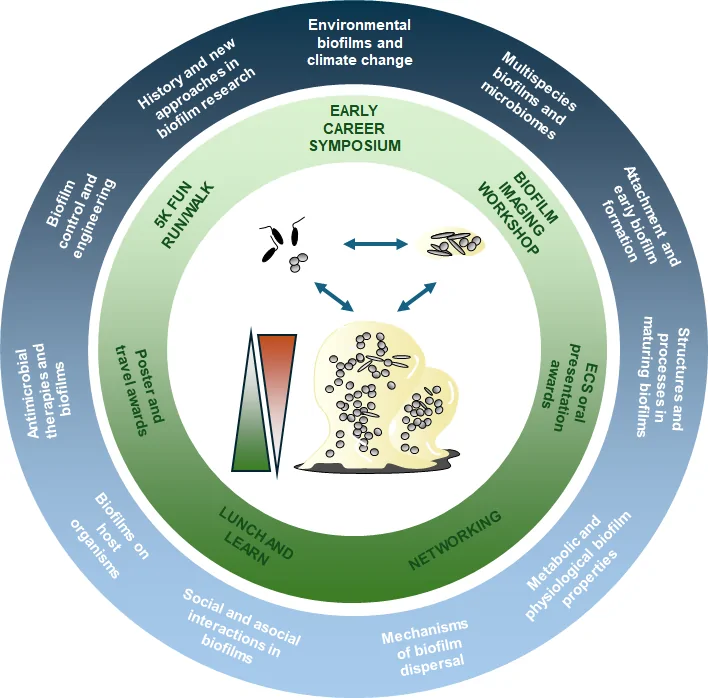
Overview of thematic sessions (outer blue circle) as they relate to biofilms, development, and heterogeneity within biofilms (indicated by triangles), and additional conference activities focusing on early-career development and social engagement (green circle). See also Boxes 1 to 3-3.

Overall, the 10th ASM Conference on Biofilms was attended by over 350 participants, with 55 full oral presentations (22 invited and 33 selected from submitted abstracts), plus 12 short lightning talks, in 11 thematic sessions. These sessions were (i) environmental biofilms and climate change; (ii) multispecies biofilms and microbiomes; (iii) attachment and early biofilm formation; (iv) structures and processes in maturing biofilms; (v) metabolic and physiological biofilm properties; (vi) mechanisms of biofilm dispersal; (vii) social and asocial interactions in biofilms; (viii) biofilms on host organisms; (ix) antimicrobial therapies and biofilms; (x) biofilm control and engineering; and (xi) history and new approaches in biofilm research (Fig. 1). Outstanding keynote talks from Matthew Parsek (University of Washington) and Nicole Dubilier (Max-Planck-Institute for Marine Microbiology, Bremen, Germany) bookended the conference. There were 187 posters presented in four separate sessions, representing an enormous breadth of exciting biofilm research. Conference chair Clay Fuqua and co-chair Karin Sauer worked together with an outstanding organizing committee composed of biofilm researchers with a wide range of expertise (Table 1) to develop the conference program.

Box 1. **LUNCH AND LEARN**

The Lunch and Learn session centered on advancing inclusive diversity with equity, access, and accountability in the microbial sciences, with a particular focus on strengthening the future workforce. Invited speaker **Jo Handelsman** (University of Wisconsin, Madison, WI, USA) delivered the keynote, “*AJEDI Makes Better Microbiology*,” outlining evidence‑based strategies for building scientific environments that foster inclusion, belonging, and equitable participation. She emphasized that such cultures not only broaden access but also enhance scientific rigor, creativity, and retention across career stages. Recognizing the need to expand early‑career engagement, particularly among undergraduates, who were underrepresented at the 9th conference, invited speaker **Caitlin Light** (Binghamton University, Binghamton, NY, USA) addressed approaches to engaging and fostering the next generation of microbiologists. In her talk, “*Cultivating Minds and Microbes: The Intersection of Education and Biofilm Research in Course‑Based Undergraduate Research Experiences*,” Light highlighted the impact of structured, hands‑on research programs, including a First‑Year Research Immersion (FRI) and biofilm‑focused REU initiatives, and that training of undergraduate students can enhance faculty research. The Early Career Symposium (ECS) committee also organized a lunch and learn event where early-career attendees could network over lunch with later-stage mentors in various career pathways.

Box 2. **Mark E. Shirtliff Biofilms Early Career Symposium**

The second Mark E. Shirtliff Biofilms ECS was held prior to the start of the main conference with the first keynote address, and brought together over 70 graduate students and postdoctoral researchers for an afternoon of science and dialogue on career journeys. The ECS was named in honor of Mark E. Shirtliff (University of Maryland, Baltimore) at its inauguration at the 9th ASM conference on biofilms (1), and support for this event at the 10th ASM conference on biofilms was provided by the Mark Shirtliff Biofilm Foundation.

Box 3. **Costerton lecture. In Memoriam of John William (Bill) Costerton**

To mark the 10th anniversary of this conference, the “Costerton Biofilm Community lecture” was introduced to honor past conference organizer **John William (Bill)** Costerton. Considered the “Father of Biofilms” and “The King of Slime”, Costerton organized the first ASM biofilm conference in 1996 in Snowbird, Utah. Bill Costerton, PhD, a pioneer in biofilm research, dedicated over 40 years to studying bacteria in biofilms. Inspired by his love for climbing in the Canadian Rockies, he first observed biofilm formation while examining slippery rocks in alpine streams. His groundbreaking biofilm research, focused on how bacteria grow within a protective matrix and adhere to solid surfaces, revolutionized our understanding of microbial life. As Director of the Center for Biofilm Engineering at Montana State University, his influence on the field of biofilm research as it matured was pervasive. Bill mentored countless students and colleagues, fostering a collaborative approach to research. Costerton published over 700 peer-reviewed papers, advancing biofilm research in environmental, dental, and medical microbiology, and leading to key medical breakthroughs in chronic and implant-related infections. The Costerton Biofilm Center at the University of Copenhagen is named in his honor, and his legacy continues to influence the field. Costerton passed away on 12 May 2012. The first invited speaker for the Costerton lecture was Karen Visick (Loyola Stritch School of Medicine), who was co-organizer of the 8th and organizer of the 9th ASM Conference on Biofilms.

**TABLE 1:** Conference organizing committee

Committee member	Institution
Clay Fuqua	Indiana University, Bloomington, IN, USA
Karin Sauer	Binghamton University, Binghamton, NY, USA
Mette Burmølle	University of Copenhagen, Denmark
Ehud Banin	Bar-Ilan University, Ramat-Gan, Israel
Birthe Veno Kjellerup	University of Maryland, College Park, MD, USA
Maria Hadjifrangiskou	Vanderbilt University, Nashville, TN, USA
Dean Rowe	Indiana University, Bloomington, IN, USA
Kendra Rumbaugh	Texas Tech University, Lubbock, TX, USA
Boo Shan Tseng	University of Nevada, Las Vegas, NV, USA
Mark Shembri	University of Queensland, Brisbane, Australia
Christophe Beloin	Pasteur Institute, Paris, France

The 2025 conference offered several of the successful features from the 2022 9th Conference on Biofilms, including Lightning Talks (Table 2) and the Mark Shirtliff Early Career Symposium. The highly successful ECS initiated the conference on the afternoon of Sunday, 9 November, organized by an Early Career Committee chaired by Caitlin Light and Megan O’Malley and composed of graduate students and postdoctoral scientists (Table 3). It featured two guest keynote talks and seven oral presentations from early-career speakers selected from the conference abstracts. Later, in the week on Wednesday afternoon (11/12), the highly subscribed Paul Stoodley Biofilm Imaging Workshop was offered. The workshop was led by Erin Gloag (Ohio State University, Columbus, OH, USA), who was assisted by William DePas (University of Pittsburgh, Pittsburgh, PA, USA), Sophie Darch (University of South Florida, Tampa, FL, USA), and Hannah Jeckel (California Institute of Technology, Pasadena, CA, USA), providing hands-on guidance on MiPACT, COMSTAT, and BiofilmQ packages for image analysis and processing, as well as for aggregate analysis.

**TABLE 2:** Lightning talks^*a*^

Presenter	Career stage	Institution	Presentation title
Rehnuma Tabassum Sejuty	Graduate student	University of Calgary, Calgary, Alberta, Canada	Heat Sensing Mechanism for a Bacterial Thermosensitive Diguanylate Cyclase
Cristina Amador	Post-doc	University of Copenhagen, Copenhagen, Denmark	Built for success: synthetic marine biofilm consortia to prevent antifouling
Megan O’Malley	Post-doc	University of Washington, Seattle, WA, USA	C-di-GMP-independent surface sensing by the Cpx envelope stress response system in early *Pseudomonas aeruginosa* biofilm development
Herby Jean-Baptiste	Graduate student	Temple University, Philadelphia, PA, USA	Genetic requirements for induction of *E. faecalis* hyperchaining and complex structure formation in biofilms
Favour Nwankpa	Graduate student	University of Binghamton, Binghamton, NY, USA	Understanding the impact of matrix proteases on the *P. aeruginosa* dispersion
Aili Wang	Graduate student	University of Wyoming, Laramie, WY, USA	Antibiotic-responsive transcription factor PssR regulates extracellular polysaccharide biosynthesis in *Listeria monocytogenes*
Sadhana Srinivasa	Graduate student	Duquesne University, Pittsburgh, PA, USA	RsmE-dependent spatial positioning manifests cooperative spreading in *Pseudomonas fluorescens* through alterations in cyclic-di-GMP levels
Nicole Levi	Associate Professor	Wake Forest School of Medicine, Winston-Salem, NC, USA	Differential damage of *Staphylococcus aureus* biofilms using photothermal nanocomposites for rapid disruption
Parker Smith	Post-doc	Georgia Institute of Technology, Atlanta, GA, USA	Precision strikes: R-pyocin killing of *Pseudomonas aeruginosa* CF isolates grown in biofilms and aggregates
Anumeha Saha	Post-doc	University of Pittsburgh, Pittsburgh, PA, USA	Genomic insights into aerosolized non-tuberculous mycobacteria from showerheads: evidence for virulence, resistance, and environmental persistence
Joselyn Molinar	Graduate student	Indiana University, Bloomington, IN, USA	Experimental evolution of the honey bee symbiont *Bombella apis* reveals a conserved two-component system implicated in host association and biofilm formation
Maria Soledad Benitez Ponce	Associate Professor	Ohio State University, Columbus, OH, USA	Spectral sensing of biofilms for controlled environment agriculture

^*a*^ Speakers are listed in order of presentation.

**TABLE 3:** Early Career Committee

Committee member	Institution	Career stage
Caitlin Light, Chair	Binghamton University, Binghamton, NY, USA	Early-career faculty
Megan O’Malley, Co-chair	University of Washington, Seattle, WA, USA	Postdoctoral scientist
Nasibeh Arabameri	University of Nevada Las Vegas, Las Vegas, NV, USA	Graduate student
Lauren Augusta	Indiana University, Bloomington, IN, USA	Graduate student
Sarah Comer	Vanderbilt University, Nashville, TN, USA	Graduate student
Angus Johnson	Dartmouth College, Hanover, NH, USA	Graduate student
Yuzhu Mao	University of Maryland, College Park, MD, USA	Graduate student
Heema Vyas	University of Adelaide, Adelaide, Australia	Postdoctoral scientist

**Fig 2:**
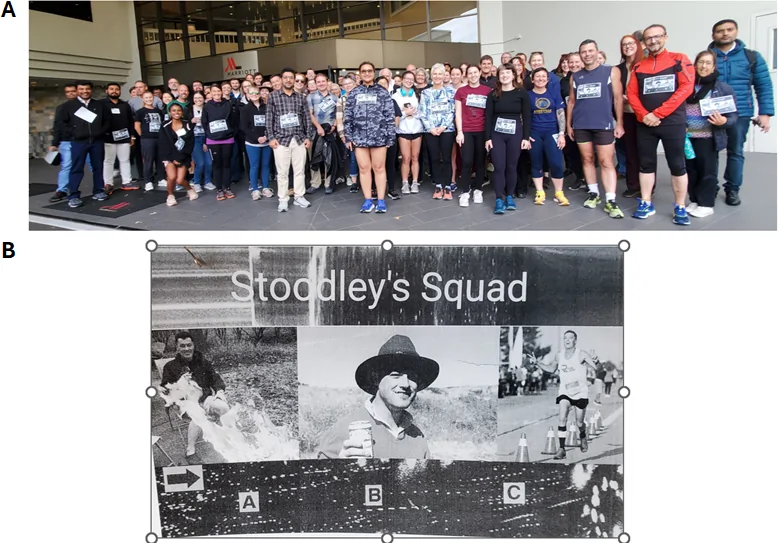
Group picture (**A**) taken at the beginning of the 5K Fun Run showing participants wearing (**B**) bibs in memory of Paul Stoodley, see also Box 4.

New features of the 2025 conference included the William J. Costerton Lecture, recognizing the amazing legacy of Bill Costerton and recognizing researchers who have contributed to the field and the conference. Also, two Lunch and Learn sessions were held (Box 1), an early-career social hour on the first night of the conference, a new mentor-mentee identification system to help connect first-time attendees or early-career scientists with mentors and conference veterans, as well as several social events including a “fun run” (Fig. 1, see also Fig. 2 and Box 4) that provided excellent opportunities for networking and collaborative interactions. Overall, these new features were considered by the conferees to be highly positive and to add value to the conference.

Box 4. **In Memoriam: Paul Stoodley, PhD**

**Paul Stoodley (1960–2024)** was a transformative leader in biofilm research whose pioneering microscopy fundamentally reshaped the field. At Montana State University’s Center for Biofilm Engineering, he advanced confocal imaging approaches that revealed the true three‑dimensional architecture of biofilms, complete with channels, mushroom‑like structures, and dynamic flow pathways, overturning long‑held assumptions and establishing principles that remain central to modern biofilm science. His more than 250 publications, along with his development of widely adopted imaging methods, influenced clinical, environmental, and engineering microbiology. Stoodley also played a pivotal role in building and sustaining the biofilm research community. He trained hundreds of scientists through hands‑on microscopy workshops, including those held at many ASM Biofilms meetings, and his service on the ASM Biofilms program committee helped shape the conference’s interdisciplinary scope. His enthusiasm for collaboration and his ability to bring people together, whether in the lab, on the ski slopes, or on a bike trail—left a lasting imprint on colleagues and trainees worldwide. The conference honored his legacy through two tributes reflecting his passions for imaging and community: a 5K fun run/walk (Fig. 2) and the dedication of the **Paul Stoodley Imaging Workshop**. These events were introduced by a remembrance from his longtime colleague **Daniel Wozniak** (Ohio State University, Columbus, OH, USA), celebrating Stoodley’s enduring impact on biofilm science and the vibrant community he helped cultivate.

## MARK SHIRTLIFF EARLY CAREER SYMPOSIUM

The ECS (Box 2), first inaugurated in 2022 in honor of Mark Shirtliff (1), continued to serve as a central forum for showcasing emerging talent and advancing the scientific and professional development of early-career researchers. The ECS was planned and organized by the Early Career Committee, chaired by Caitlin Light and Megan O’Malley, and composed of graduate students and postdoctoral scientists (Table 3), and this symposium kicked off the conference. The program blended cutting-edge science with candid reflections on personal and professional growth, reinforcing that scientific progress and the sustainability of the scientists are inseparable, through two keynote lectures and seven talks selected from the abstracts, highlighting both conceptual advances in biofilm biology and the diverse trajectories that shape scientific careers. Keynote speakers were **Dominique (Nicki) Limoli** (Indiana University, Bloomington, IN, USA), who presented her research on the coinfection and attraction of *Pseudomonas aeruginosa* and *Staphylococcus aureus* in the cystic fibrosis lung environment, and **Thomas Seviour** (Aarhus University, Aarhus, Denmark), who spoke about biofilm matrix assembly, specifically eDNA, and how understanding biofilm matrix assembly and regulation can be leveraged to improve biofilm control in clinical and industrial (e.g., wastewater treatment) settings. Both speakers emphasized the nonlinear nature of scientific careers, that each life experience teaches and guides us to our next steps, and brings us closer and closer to our passion and fulfillment. Nicki also challenged ECS attendees to think about sustainable science from a new perspective: the sustainability of the scientists and human capital of the research endeavor, such as resources required for maintaining research productivity and enthusiasm, including pursuits like sleep, family, friends, exercise, and more.

Selected scientific presentations by graduate students and postdoctoral researchers reflected the field’s rapid methodological expansion. Three talks (Huda Usman, Carnegie Mellon University, Pittsburgh, PA, USA; Jessica Li, University of Michigan, Ann Arbor, MI, USA; and Kathryn Zimlich, Montana State University, Bozeman, MT, USA) introduced innovative engineering platforms, including nanoculture systems and 3D bioprinting to interrogate biofilm growth, structure, and emergent properties. Two additional presentations leveraged single-cell imaging and transcriptomics to resolve heterogeneity within biofilms, revealing lineage-specific behaviors and regulatory clusters obscured in bulk analyses. Jung-Shen Benny Tai (Laboratory of Jing Yan, Yale University, New Haven, CT, USA) presented imaging and lineage-tracking approaches integrated with agent-based modeling to examine phenotypic variability during biofilm development, revealing that spontaneous heterogeneity can determine positional cell fate. Nasibeh Arabameri (Laboratory of Boo Shan Tseng, University of Nevada, Las Vegas, NV, USA) applied single-cell RNA sequencing to map transcriptional diversity, identifying regulatory clusters obscured in bulk analyses. Spatial localization using HCR-FISH further clarified how gene-expression heterogeneity shapes biofilm structure and function.

Mechanistic insights were further advanced through studies of temperature-responsive diguanylate cyclase activity in *Pseudomonas aeruginosa* (Rehnuma Tabassum Sejuty, Laboratory of Joe Harrison, University of Calgary, Calgary, Alberta, Canada) and an uncharacterized lysis-sensing pathway in *Vibrio parahaemolyticus* where a kin-specific metabolite turns kin death into collective protection by driving motility-based aggregation (Yangyang Liu, Laboratory of Andrew Bridges, Carnegie Mellon University, Pittsburgh, PA, USA). Liu and Arabameri were selected by the Early Career Committee to present their work during the main conference program (Table 4).

**TABLE 4:** Conference award recipients^*a*^

Awardee	Institution	Sponsor	Award
Amyx-Sherer, Kristen (GS)	Washington University of St. Louis, St. Louis, MO, USA	Mark Shirtliff Biofilm Foundation	Poster award
Arabameri, Nasibeh (PD)	University of Nevada Las Vegas, Las Vegas, NV, USA	ASM, EC	Travel award, EC oral presentation award
Barrington, Marshall (GS)	Washington University of St. Louis, St. Louis, MO, USA	ASM	Travel award
Benedetti, Stefania (PD)	University of Hospital Bonn, Bonn, Germany	ASM	Travel award
Carpenter, Carrie (GS)	Virginia Tech. Blacksburg, VA, USA	ASM, ASM journals	Travel award, poster award,
Dearing, Hailey (GS)	University of Washington, Seattle, WA, USA	Mark Shirtliff Biofilm Foundation	Poster award
Deaver, Stacie (GS)	Virginia Tech, Blacksburg, VA, USA	ASM journals	Poster award
Eck, Frederik (GS)	Justus-Liebig-University, Giessen, Germany	ASM	Travel award
Fonteyne, Flor (PD)	KU Leuven, Leuven, Belgium	ASM	Travel award
Fu, Yu (GS)	Dartmouth College, Hanover, NH, USA	ASM	Travel award
Fujita, Mao (GS)	University of Tsukuba, Tsukuba, Ibaraki, Japan	ASM	Travel award
Geppert, Andrew (GS)	University of Calgary, Calgary, Alberta, Canada	ASM	Travel award
Hansen, Mads Fredrik (GS)	University of Copenhagen, Copenhagen, Denmark	ASM	Travel award
Holmes, Patra (GS)	Columbia University, New York, NY, USA	ASM	Travel award
Hunt, Benjamin (PD)	University of Buffalo, Buffalo, NY, USA	ASM, ASM journals	Travel award, poster award
Isenberg, Ruth (PD)	University of Minnesota, Twin Cities, MN, USA	ASM	Travel award
Johnson, Angus (GS)	Dartmouth College, Hanover, NH, USA	ASM	Travel award
Kanno, Mizuki (GS)	Graduate School of Science and Technology, Shizuoka University, Shizuoka, Japan	ASM	Travel award
Kataoka, Masahito (GS)	University of Tsukuba, Tsukuba, Ibaraki, Japan	ASM	Travel award
Li, Xiaolei	University of Pennsylvania, Philadelphia, PA, USA	ASM journals	Poster award
Liu, Yangyang (GS)	Carnegie Mellon University, Pittsburgh, PA, USA	ASM, EC	Travel award, EC oral presentation award
Mao, Yuzhu (GS)	University of Maryland, College Park, MD, USA	ASM	Travel award
Nomura, Keisuke (GS)	University of Tsukuba, Tsukuba, Ibaraki, Japan	ASM	Travel award
Sejuty, Rehnuma Tabassum (GS)	University of Calgary, Calgary, Alberta, Canada	ASM, FEMS journals	Travel award, poster award
Wucher, Benjamin (PD)	Memorial Sloan Kettering Cancer Center, New York, NY, USA	ASM journals	Poster award

^*a*^ (Career Stage—GS, graduate student; PD, post-doc), EC, Early Career Committee; ECS, Early Career Symposium.

The ECS was praised for its added value to early-career scientists at the ASM Conference on Biofilms, reinforcing its role as a catalyst for scientific innovation, community building, and professional inspiration. Attendees consistently noted the symposium’s contribution to the supportive and energetic tone of the ASM Biofilms Conference, underscoring its growing impact on the early-career microbiology community. This is apparent by Nasibeh Arabameri’s feedback, “*When I was in college back in Iran, I dreamed of being part of the biggest microbiology community in the world—ASM. Ten years ago, it felt completely out of reach. I never imagined that one day I wouldn’t just be an ASM member, but that I’d actually be presenting my research at one of their most prestigious conferences, ASM Biofilms…I received such thoughtful feedback and left every session inspired by the science and the people in this community…This conference has always been one of my favorites, and this year reminded me exactly why*.”

## KEYNOTE ADDRESS: MATTHEW PARSEK

**Fig 3:**
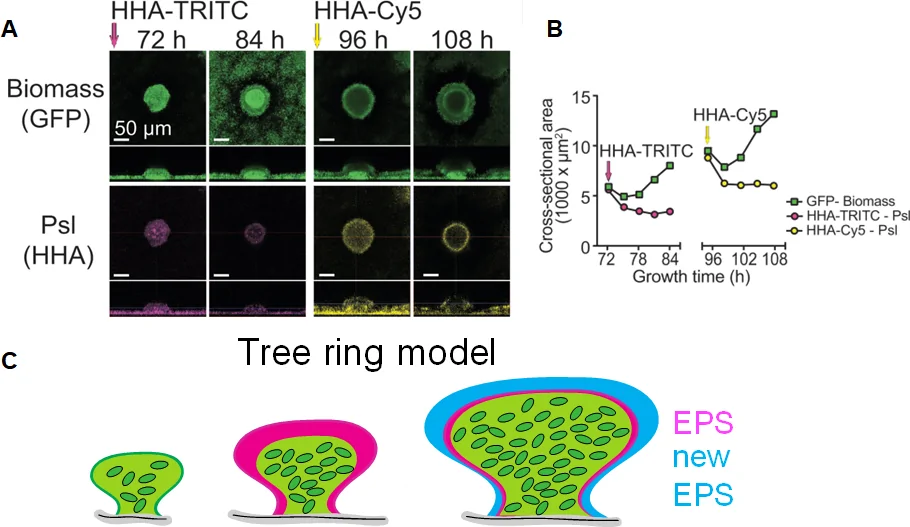
The ”tree ring” model of matrix dynamics. New Psl is continually deposited at the aggregate periphery as a biofilm aggregate grows, with older matrix material being continually turned over. This dynamic pattern has been dubbed the tree ring model. (**A**) Flow cell biofilms were labeled at different timepoints with fluorescently conjugated Psl-specific lectins and imaged by confocal microscopy. Shown is a typical aggregate observed at this time point. Fluorescence due to GFP is shown in green, HHA-TRITC is shown in magenta, and HHA-Cy5 is shown in yellow. (**B**) The cross-sectional areas of biofilm biomass and Psl observed by microscopy were measured, and the values indicate that Psl does not migrate to the aggregate periphery, but that new Psl is deposited at the aggregate periphery. (**C**) As aggregates grow over time (depicted from left to right in the diagram), existing matrix material is turned over, and new matrix is deposited at the aggregate periphery. Image kindly provided by M. Parsek, reproduced with permission (2).

**Matt Parsek** opened the conference with a keynote that traced the long history and personal significance of this meeting and biofilm science from its origins in the early Costerton-era meetings (Box 3) to the sophisticated, mechanistic discipline it has become. He emphasized how decades of work have overturned the early view of biofilms as simple surface-associated layers, revealing instead highly structured, heterogeneous communities shaped by spatially organized matrix components and multilayered regulatory networks. Using *P. aeruginosa* as a model, Dr. Parsek synthesized his group’s contributions to defining the composition, spatial organization, and functional dynamics of the biofilm extracellular matrix, highlighting stratified production of the exopolysaccharides Pel, PSL, and alginate, as well as extracellular DNA (eDNA) and the adhesin CdrA. He emphasized that their expressions differ across strains and are highly stratified within biofilm aggregates. Using genetic tools, fluorescent reporters, and inducible-expression systems, his group discovered that matrix production is not uniform: new matrix is selectively synthesized and deposited at the periphery of growing aggregates while older matrix remains centrally retained, supporting a “tree-ring” model of biofilm growth rather than earlier balloon-like models (2) (Fig. 3). A major focus of the Parsek lab has been on CdrA, a large, multifunctional matrix protein that binds Pel and PSL polysaccharides and is essential for aggregate expansion and architectural integrity (3–6). Remarkably, CdrA persists in the matrix long after its production ceases, functioning both in its cell-associated form and as a released extracellular crosslinker, contributing to biofilm stability even when no longer synthesized. These structural principles appear conserved across multiple species and have been validated in clinical specimens such as cystic fibrosis sputum, underscoring their physiological relevance (7). Parsek further integrated advances in understanding the regulatory pathways of biofilm initiation and maturation, detailing how quorum-sensing, cyclic AMP (cAMP)-dependent motility pathways, and cyclic-di-GMP (c-di-GMP) signaling interact to drive early differentiation and matrix expression. Single-cell reporter studies revealed that bacteria rapidly diverge into high- and low-cyclic-di-GMP subpopulations upon surface contact (8), and the physical environment profoundly shapes these signaling trajectories: flow-cell systems consistently induce cyclic-di-GMP production, while agar–air interfaces preferentially activate cAMP-mediated pathways (9). Experiments manipulating viscosity and stator function further revealed that swimming speed strongly influences signaling outcomes, supporting a model wherein fast-swimming or polarly tethered cells activate SadC-dependent cyclic-di-GMP signaling during surface sensing (9). The talk concluded with emerging data comparing spatial and temporal patterns of c-di-GMP and quorum-sensing activity, raising new questions about how multiple signaling networks are coordinated during biofilm development. Overall, the keynote synthesized decades of research to illuminate the structural, regulatory, and biophysical principles that govern biofilm formation and persistence.

## SESSION 1: ENVIRONMENTAL BIOFILMS AND CLIMATE CHANGE

As the first session of the week-long conference, one of the Grand Challenges of our time—Climate Change—was the overall topic from impacts of rising temperatures in Antarctica, to degradation of fossil-fuel-derived plastics, to meandering rivers. The invited speakers for this session were **Christine Foreman** (Montana State University, Bozeman, MT, USA) and **Judy Q. Yang** (University of Minnesota, Twin Cities, MN, USA). Foreman presented studies on *Serratia* sp. PL17, isolated from Pony Lake, Antarctica, a biofilm-forming, cold-temperature-adapted bacterium. The Foreman lab has developed *Serratia* sp. PL17 as a genetic model system and identified the genes for a cold-adapted biosurfactant that improves long-chain hydrocarbon utilization at cold temperatures. Subsequent studies have shown that this bacterium plays a key ecological role, where biosurfactant production in biofilms led to increased survival and biodegradation activity in water as well as sediment interfaces, thus making bioremediation of hydrophobic hydrocarbons a real possibility in cold climates. Yang presented her lab’s work examining how fluid flow dynamics and microscale roughness on particles can influence mass transfer processes at surfaces. Biofilms present on sediment particles in rivers and other aquatic environments can change the mass transfer at the macroscale, but many current sediment models do not include these quantitative processes. The Yang lab is using microfluidic devices to examine how these processes influence biofilm formation over time and as a result of different shear stress. In addition, macroscale flumes are used to simulate river flow, providing insights into the role of biofilms in erosion and geomorphology, and explaining how biofilms on sediment particles can influence the way that a river meanders at the macroscale.

Also in this session, **Bin Cao** (Washington State University, Pullman, WA, USA) presented his group’s work on the degradation of environmental plastics using an “artificial worm gut” biofilm community derived from plastic-eating superworms (10). Whole plastic degradation pathways encoded by many degradative genes within the “artificial worm gut” are now recognized as one potential solution to the problem of plastic pollution. The impact of phototrophic biofilms on stone monuments was described by **Isaac Klapper** (Temple University, Philadelphia, PA). These communities cause discoloration and damage to the monuments, and these processes may be exacerbated by the increased atmospheric CO_2_. Klapper described how periodic saturation and humidity also contribute to these processes. Finally, in this session, **Hannah Jeckel** (Laboratory of Diane Newman, California Institute of Technology, Pasadena, CA, USA) described her work on a plant rhizosphere-associated, six-member synthetic community. One of the community members, *Pseudomonas synxantha,* produces phenazines, which can have profound effects on the composition of the community. Observations across different experimental setups, including a phytagel-based system to cultivate wheat plants, are combined to investigate the impact of the environment on microbial interactions, for example, the effect of diffusible toxins such as phenazines on the community.

## SESSION 2: MULTISPECIES BIOFILMS AND MICROBIOMES

Using diverse systems ranging from biocrusts, phage-protected synthetic communities, and polymicrobial urinary tract biofilms, this session on multispecies biofilms and microbiomes revealed how multispecies organization fundamentally reshapes biofilm function. Features including stability, resilience, and biofilm behavior arise from cooperative and antagonistic interactions within structured microbial consortia.

**Fig 4:**
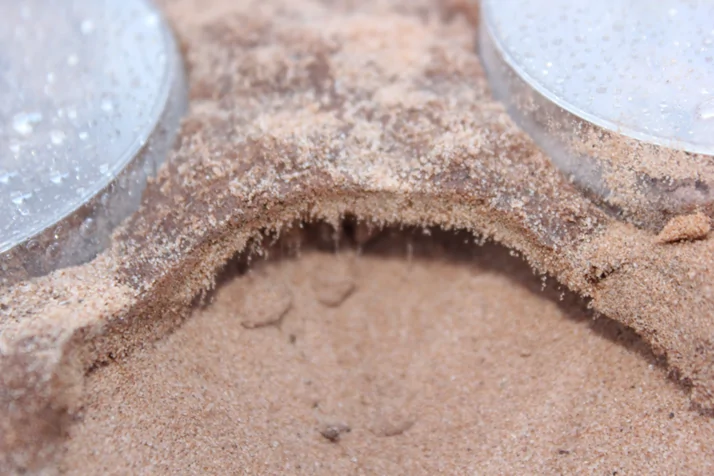
Close-up view of a cryptic biocrust on sandy soils in South-eastern Utah after sampling with a Petri dish. The biofilm, built by the cyanobacterium *Microcoleus vaginatus,* holds the otherwise loose soil against gravity, with clusters of sand grains visibly attached to its (here invisible) filaments. Petri dishes are 5 cm in diameter. Image kindly provided by **F. Garcia-Pichel**.

The session opened with invited speaker **Ferran Garcia-Pichel** (Arizona State University, Phoenix, AZ, USA), who presented work on microbial interactions within desert biological soil crusts (Fig. 4), dry phototrophic biofilms that drive carbon and nitrogen fixation and support soil stabilization. Garcia-Pichel focused on the cyanobacterium *Microcoleus vaginatus*, a dominant pioneer organism in biocrusts. Although critical for biocrust formation, *M. vaginatus* is unable to fix nitrogen and fails to establish in monoculture during restoration efforts, highlighting its reliance on metabolic interactions with other bacterial partners. His work further showed that *M. vaginatus* relies on compatible nitrogen-fixing partners for establishment, while antagonists, including the globally distributed obligate cyanobacterial predator ***Candidatus* Cyanoraptor togatus,** which invades and kills *M. vaginatus*, destabilize biocrust structure. Destabilization results in loss of nitrogen fixation, compromised dust capture, and moisture retention, highlighting the delicate balance of facilitation and antagonism that underpins these communities, and underscoring the emergent ecological functions of biocrusts.

Building on the importance of spatial structure, **Mads Frederik Hansen** (Laboratory of Mette Burmølle, University of Copenhagen, Copenhagen, Denmark) examined how community architecture shapes bacterial survival during phage exposure. Using a synthetic biofilm community composed of *Escherichia coli*, *Kluyvera cryocrescens*, and *Vibrio anguillarum*, he showed that *E. coli,* normally significantly reduced by the T7 phage, survived when embedded within the multispecies community. This protection was specifically dependent on matrix components produced by *V. anguillarum,* as mutants lacking matrix components (*vpsMNOP* or *rbmC*) formed less compact biofilms that no longer shielded *E. coli*. High-resolution imaging confirmed that spatial packing, rather than phage adsorption or biomass, was the key determinant of protection. These findings demonstrate that community architecture can generate collective phage tolerance. The session then shifted to the oral microbiome, where **Bruno Lima** (University of Florida, Gainesville, FL, USA) investigated how protein acetylation regulates *Streptococcus gordonii* biofilm formation. The acetyltransferases SktA and SktB were strongly upregulated in biofilms, but only *sktA* deletion altered architecture, producing thin, poorly structured biofilms with reduced EPS. In a mouse model, the *sktA* mutant showed impaired colonization, linking SktA-dependent matrix formation to *in vivo* fitness. Proteomics identified 59 acetylated proteins, including 25 that were dependent on SktA, highlighting broad regulatory roles. Finally, **Steven Taddei** (Laboratory of Chelsie Armbruster, University of Buffalo, Buffalo, NY, USA) explored interactions among *Enterococcus faecalis*, *Escherichia coli*, and *Proteus mirabilis*, pathogens frequently co-isolated from catheter-associated urinary tract infections. While most pairings showed limited synergy, triple-species biofilms displayed markedly increased biomass and elevated matrix protein content. Clinical isolates exhibited similar trends, particularly in chronic infections. Early *E. coli* colonization promoted subsequent community assembly and conferred ciprofloxacin protection, underscoring how polymicrobial interactions drive robust biofilm formation and emergent antibiotic tolerance.

Invited speaker **Joao Xavier** (Memorial Sloan Kettering Cancer Center, New York, NY, USA) described his transition from biofilm research to clinical oncology and highlighted the largely unexplored connections among biofilms, the gut microbiome, immunity, and cancer. He emphasized that ecological principles such as alpha and beta diversity offer a useful framework for understanding and managing microbiome dynamics in clinical settings. This was illustrated through a study of hematopoietic transplant patients in which prophylactic antibiotics caused massive drops in microbiome diversity, which predisposes patients to worse outcomes. During the 35-day period of profound immune deficiency, longitudinal fecal sampling and 16S rRNA profiling revealed that patients with higher baseline microbial diversity had a fivefold increase in long-term survival. Conversely, patients with reduced diversity but often enriched in facultative pathogens capable of exploiting immune dysfunction showed poorer outcomes. To extend these findings, Xavier’s team developed a machine-learning model incorporating data from more than 1,000 patients and 5,000 samples. The model predicts that microbiome recovery accelerates neutrophil and white blood cell reconstitution and identifies specific microbial groups associated with improved immune recovery.

## SESSION 3: ATTACHMENT AND EARLY BIOFILM FORMATION

During biofilm formation, microbes integrate mechanical cues, chemical gradients, and surface structures, utilizing specialized adhesins and regulatory networks to initiate spatially organized communities. This session focused on these complex interconnected processes and their orchestration during surface colonization.

The session opened with invited speaker **Andrew (Drew) Bridges** (Carnegie Mellon University, Pittsburgh, PA, USA), who presented an integrated analysis of biofilm dynamics and cell fate in *Vibrio cholerae*. Bridges introduced a non-disruptive brightfield-imaging approach (label-free analysis of biofilms, LFAB) that accurately captures biofilm development across multiple species, with results consistent with confocal microscopy. Extensive time-lapse data sets of *Vibrio cholerae* biofilm formation—approximately 150 movies per strain—were used to train a classifier capable of distinguishing different morphological trajectories. Scaling this strategy to 3,000 transposon mutants enabled functional clustering of phenotypes linked to motility, biotin biosynthesis, O-antigen production, and quorum-sensing pathways. A complementary high-throughput screen of 13,000 compounds to identify molecules that perturb these defined behavioral signatures is now underway. In the second part of his talk, Bridges described the use of a far-red fluorogen-activating protein to label *V. cholerae* under anoxic conditions, allowing visualization of a subpopulation of non-dispersing cells retained within the biofilm after dispersal. RNA-seq revealed a transcriptional program distinct from that of dispersing cells, and these retained cells exhibited heightened tolerance to antibiotics, oxidative stress, and phage predation. Their surrounding matrix persisted rather than being recycled, suggesting a specialized protective niche. Ongoing work aims to determine how these non-dispersing cells are specified and how divergent cell fates shape biofilm evolution. Focusing on a less heavily studied biofilm model, invited speaker **Karen Ottemann** (UC Santa Cruz, Santa Cruz, CA, USA) presented on the multiple functions of flagella to control biofilm formation by the gastric pathogen *Helicobacter pylori. H. pylori* is a microorganism for which long-term colonization of the stomach is associated with pyloric ulcers and cancers. Motility behaviors are essential for this pathogen to thrive in the gastric environment, notably to move within mucus layers and colonize the stomach epithelial layer. The flagellar system of *H. pylori* is complex, and it is integrated with additional type IV pili surface appendages (11). Ottemann described an intriguing connection between proteins that function as pilus components, PilO, PilM, and PilN, which are now recognized to form a cage around the flagellar motor. Additional flagellar components also interact with the PilM component of this cage, suggesting that the structure of the *H. pylori* flagellar apparatus is much more complex than expected based on other systems, perhaps reflecting the novel requirements of motility through gastric mucus. Further analysis of multiple flagellins suggests that chimeric filaments are even more complex because they are sheathed. The functions for these flagellins in biofilm formation are still uncertain, and additional work is necessary to elucidate their role.

*V. cholerae* utilizes the mannose-sensitive hemagglutinin (Msh) type IV pili in surface attachment, with the Msh basal body also serving as a target for c-di-GMP control of biofilm formation. **Kyle Floyd** (Illinois State University, Normal, IL, USA) focused on the function and expression of the *msh* genes encoded in two large operons, *msh-I* (*mshHIJKLMNEGF*) and *msh-II* (*mshBACDOPQ*). Systematic analysis of independent *msh* mutants revealed multiple genes required for pilus formation and five genes (*mshH*, *F*, *M*, *B,* and *Q*) that are not required, but probably modulate pilus activity. Transcription of the *msh-I* and *msh-II* operons originates from multiple promoters, suggesting complex regulation for the production of these multifunctional appendages. Deciphering the various actors for this regulation could prove useful for the development of novel anti-cholera strategies. Two of the talks in this session focused on plant-associated bacteria. **Gail Hardy** (Laboratory of Clay Fuqua, Indiana University, Bloomington, IN, USA) described her findings on the molecular basis of unipolar polysaccharide (UPP) cell surface anchoring required for surface attachment and biofilm formation in *Agrobacterium tumefaciens*. UppF, annotated as an O-antigen ligase, was found to be required to tether UPP at the cell pole. Deletion mutants of *uppF* are unable to attach to surfaces, and although they produce UPP, this is shed from cells, suggesting an anchoring defect. UPP can be isolated using a lipopolysaccharide (LPS) purification protocol consistent with a covalent linkage between UPP and LPS. Remaining to be determined are the site of UPP attachment within the LPS structure, whether unipolar localization of UPP production reflects spatial regulation of UppF within the cell envelope, and the biomechanical properties conferred by this newly recognized LPS–UPP composite. The plant association of *Pseudomonas protegens* on plant roots was described by **Guy Sobol** (Laboratory of David Hershey, University of Wisconsin, Madison, WI, USA) using a Tn-Seq approach and a novel model of colonization with viable detached roots, to identify genetic determinants of early attachment. The most dramatically impacted mutants for root colonization were disrupted in the Gac regulatory system, found in many other host-associated pathogens. The Gac sensing system was revealed to be activated upon root attachment, in response to root exudates and the drag on flagellar rotation. This suggests that the Gac system has a dual sensory role in this system, detecting both mechanical and chemical signals to promote root colonization.

## SESSION 4: STRUCTURES AND PROCESSES IN MATURING BIOFILMS

Mature biofilms modify their own environment and create spatial organization and structure in surface-associated microbial populations. This session revealed new functions of previously recognized structural and regulatory determinants of maturing biofilms. Presentations highlighted a calcium-responsive sensor that shapes early biofilm architecture in response to environmental cues, and other moonlighting transcriptional regulators that regulate biofilm structure. Cell surface features were also a theme, including how OprF governs biofilm maintenance by coordinating eDNA release and envelope stress responses, and phospholipids function as bona fide matrix components. The combined forces of biofilm mechanics and mixed-species interactions were also reported to critically drive persistence in biofilms.

**Karen Visick** (Loyola Stritch School, Medicine, Maywood, IL, USA) delivered the inaugural Costerton Lecture (Box 3), which highlighted how the bioluminescent symbiont *Vibrio fischeri* integrates environmental cues to regulate biofilm formation, leading to light organ colonization of the Hawaiian bobtail squid *Euprymna scolopes* (12). Central to this process is the *syp* gene cluster, which encodes the machinery for producing the symbiosis polysaccharide (SYP), a matrix component essential for cell–cell adhesion. Visick emphasized how calcium acts as a key external signal that promotes *V. fischeri* biofilm formation, including cellulose production through the CasA diguanylate cyclase and VpsR and SYP production through an unknown pathway (13, 14). Control of SYP production is complex, with input from multiple two-component regulators, including the sensor kinase SypF, which activates two downstream response regulators, SypG and SypE. SypF integrates opposing signals from three additional sensor kinase/phosphatases: BinK represses its activity, whereas RscS and the nitric-oxide-responsive HahK act as inducers. Loss of BinK enhances calcium responsiveness, revealing its role as a gatekeeper of SypF activation. Although RscS is a well-established positive regulator of *syp* transcription, Visick provided evidence that it is dispensable for calcium-induced biofilms, a notable departure from its important role under other conditions. Current work is probing the possibility that SypF directly senses environmental calcium to modulate *syp* transcription and control SYP-dependent biofilm development in *V. fischeri*.

Shifting from symbiotic interactions in invertebrates to human lung infections, Invited Speaker **Luanne Hall-Stoodley** (Ohio State University, Columbus, OH, USA) presented new insights into *Mycobacterium abscessus* (MAB) biofilm biology and its interactions with *P. aeruginosa* which can be isolated from the airways of people with cystic fibrosis (pCF). MAB is an emerging nontuberculous mycobacterial pathogen that causes highly persistent pulmonary infections in people with CF, who are over 1,000-fold more susceptible to nontuberculous mycobacteria (NTM) colonization. Hall-Stoodley emphasized the clinical importance of MAB morphotypes: although both smooth and rough variants form biofilms, the rough variant is more virulent, more refractory to eradication, and associated with accelerated lung function decline. Using primary human differentiated CF bronchial epithelial cultures, the rough variant exhibited significantly greater epithelial adherence, hyperaggregation in mucus, and induction of mucus hypersecretion, suggesting a feed-forward cycle that promotes chronic infection. Rheological analyses revealed that although both variants formed biofilms that behaved as viscoelastic fluids, rough-variant biofilms were stiffer, suggesting increased resistance to mucociliary and cough-mediated clearance compared with *P. aeruginosa* biofilms. In dual-species biofilm models, *P. aeruginosa* antagonized MAB, reducing its abundance by 2–3 logs, an effect absent in planktonic co-culture. Surprisingly, antagonism persisted across *P. aeruginosa* mutants defective in contact-dependent (T3SS and T6SS) and contact-independent (quorum-sensing and type II secretion) pathways, suggesting a previously unrecognized antibacterial mechanism. Transcriptomic profiling of co-cultured biofilms revealed coordinated stress responses in both organisms. These findings highlight how biofilm mechanics and interspecies interactions may shape the persistence of MAB in CF airways and reveal new molecular signatures of antagonism and stress adaptation within mixed-species biofilms.

In addition to the mixed bacterial infections, such as the MAB-*P. aeruginosa* interaction illustrated by Hall-Stoodley, *P. aeruginosa* also interacts with viral pathogens during lung infections. **Lia Michaels** (Laboratory of Jennifer Bomberger, Dartmouth College, Hanover, NH, USA) presented new insights into how co-infection with respiratory syncytial virus (RSV) modulates *P. aeruginosa* biofilm formation. Surprisingly, this modulation is through the type VI secretion (T6SS) immunity protein TsiT, previously recognized for its protection of *P. aeruginosa* from T6SS-mediated toxification. RSV infection of bronchial epithelial–biofilm cocultures enhanced *P. aeruginosa* biofilm formation and increased iron availability, driving upregulation of the T6SS TseT operon, including TsiT. Using epithelial and microfluidic flow-cell models, Michaels showed that deletion of the TseT operon impaired biofilm growth, whereas ectopic expression of *tsiT* restored biomass accumulation. Structural homology and DNA-binding assays revealed a previously unrecognized DNA-binding function for TsiT, dependent on a predicted DNA-binding domain. RNA-seq of a *tsiT* mutant indicated that TsiT activates pathways to promote biofilm formation, acting as a moonlighting transcriptional regulator linking T6SS and biofilm formation, as well as serving as a nexus for viral and bacterial interactions in lung infections. A different major biofilm factor in *P. aeruginosa* was the focus of **Erin Cassin** (Laboratory of Boo Shan Tseng, University of Nevada, Las Vegas NV, USA), who presented new findings on how the abundant outer-membrane porin OprF supports biofilm maintenance by coordinating eDNA release, proteolysis, and envelope-stress responses. OprF mutants (∆*oprF*) were diminished for static culture biofilm formation, and coinciding with ~60% less eDNA, consistent with decreased cell lysis. Transcriptomic profiling of static biofilms of the ∆*oprF* mutant identified hundreds of differentially expressed genes, including upregulation of matrix-associated proteases, and decreased expression of genes involved in lysis-mediated eDNA release and stress-related sigma factor genes. These data suggest that OprF may help sense or respond to envelope stress in *P. aeruginosa* biofilms, with OprF playing a regulatory role in mature biofilms, rather than promoting biofilm formation through canonical exopolysaccharide or vesicle production pathways. Highlighting another unrecognized contributor to matrix formation, **Shinya Sugimoto** (Jikei University School of Medicine, Minato, Japan) presented evidence that membrane phospholipids are integral structural components of *Staphylococcus aureus* biofilms, expanding the classical view of the matrix beyond eDNA, proteins, and polysaccharides. Using biochemical extraction and the instantaneous clearing of biofilm (ICBiofilm) assay (15), a whole-biofilm clearing and imaging method that enables live and dynamic imaging of biofilm development, Sugimoto identified extracellular lysyl-phosphatidylglycerol (Lys-PG), phosphatidylglycerol, and cardiolipin within mature biofilms. Treatment with phospholipase A selectively dispersed established biofilms and inhibited their formation without affecting viability, implicating membrane-derived phospholipids in matrix integrity. Functional assays revealed that Lys-PG, but not cardiolipin, promoted cell aggregation, with Lys-PG localized to the cell surface, where its positive charge and hydrophobic tail facilitate cell aggregation independent of proteinaceous adhesins. Disruption of *mprF*, required for Lys-PG synthesis, reduced attachment and yielded thinner biofilms, confirming its physiological relevance.

## SESSION 5: METABOLIC AND PHYSIOLOGICAL BIOFILM PROPERTIES

The spatial patterning of heterogeneous physiological states within the multicellular biofilm structure has long fascinated researchers. This session fostered discussion of this phenomenon across prokaryotic and eukaryotic systems, with an emphasis on metabolic exchange and cooperation between distinct biofilm subpopulations, and biofilm behavior arising from the interplay of environmental gradients, metabolic specialization, and structural patterning.

Invited speaker **Lars Dietrich** (Columbia University, New York, NY, USA) opened the session with a detailed examination of the three-dimensional chemical and metabolic architecture of *P. aeruginosa* PA14 biofilms. The Dietrich Lab uses fluorescent reporter strains and, in collaboration with Wei Min’s group (also at Columbia), stimulated Raman scattering microscopy to examine bacterial gene expression and metabolic activity. High-resolution imaging of thin-sectioned biofilms, combined with oxygen-sensing probes, revealed steep oxygen gradients that structure metabolism. In the anoxic interior, pyruvate fermentation generated D-lactate that diffused upward to oxic layers, where it was reutilized as a carbon source, effectively recycling pyruvate. Other loci involved in anaerobic metabolism mirrored these gradients: *napA* was expressed in oxic regions, whereas *nirS* expression localized to deeper anoxic zones (16). Cell organization also varied sharply with oxygen availability; randomly arranged cells in anoxic zones contrasted with vertically striated arrangements in oxic regions, a pattern dependent on O-antigen biosynthesis (*ΔwbpM* [17]). Dietrich followed his findings of biofilm microanatomy by demonstrating that differences in cell arrangement have physiological consequences. While biofilms formed by wild-type PA14 demonstrated maximum metabolic activity in the center of the striated oxic zone, a zone that was most affected by tobramycin treatment, these zones were shifted into the upper layers of the disorganized ∆wbpM biofilm. Dietrich further showed that glycolate, produced in part via the glyoxylate shunt and enriched in oxic layers, binds to the lactate regulator LldR and represses lactate metabolism, providing a regulatory link between metabolite exchange and physiological stratification in biofilms (18).

**Fig 5:**
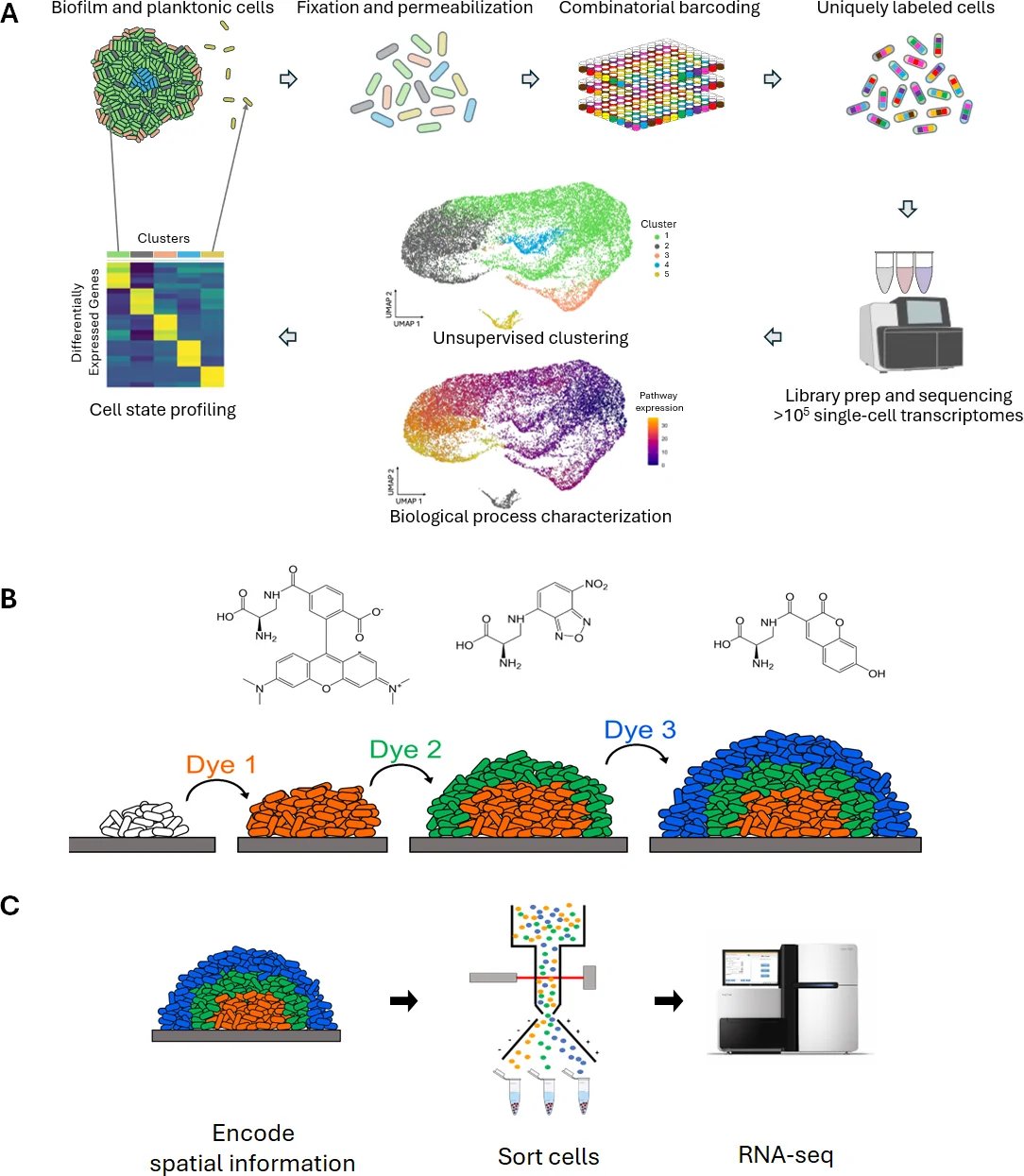
Overview of two scRNA-seq approaches. (**A**) MicroSPLiT is a scRNA-seq method for bacteria. The method is based on a split-pool barcoding system by which each cDNA within a cell receives a unique barcode combination, which is used as cell identity. The steps in the process are shown and include permeabilization and fixation of cells, three rounds of barcoding using RT and ligation process, cell lysis, library preparation, and sequencing. (**B and C**) Overview of RAINBOW-Seq. (**B**) Growth-based spatial encoding with fluorescent dyes that only stain the growing bacteria at the biofilm periphery. (**C**) The fluorescence staining is used to flow-sort biofilm cells by radial position before RNA-seq. Images kindly provided by S. Fedak (**A**) and J. Liu (**B and C**).

Echoing the spatial themes, **Angus Johnson** (Laboratory of Robert Cramer, Dartmouth Geisel School of Medicine, Hanover, NH, USA) described analogous vertical architectures in *Aspergillus fumigatus*, where the furrowed “H-MORPH” colony morphology supports enhanced growth under anoxic conditions (19). Genetic analyses identified the *baf* gene family and the global regulator LaeA as key determinants of vertical extension and extracellular polysaccharide production, illustrating how population-level morphology reflects underlying physiological strategies relevant to infection (20–22). Advancing single-cell resolution, **Stephen Fedak** (Laboratories of Anna Kuchina and James Carothers, Institute for Systems Biology and University of Washington, Seattle, WA, USA) applied microSPLiT (microbial split-pool ligation transcriptomics, Fig. 5A) (23) to map transcriptional heterogeneity in *P. aeruginosa* PAO1 across planktonic, surface-attached, and aggregate-forming conditions. Pseudotime trajectory analysis is a computational method used in single-cell transcriptomics to reconstruct dynamic biological processes by inferring sequential changes in gene expression, even when the data come from a single snapshot in time (24). Pseudotime trajectories revealed transitions from motility-associated early states to metabolically reprogrammed biofilm states, with the ANR transcription factor and the small RNA *sicX* emerging as regulators of the planktonic-to-biofilm shift. **Kailyn Jessel** (University of Michigan, Ann Arbor, MI, USA) extended the theme of spatial differentiation to *E. coli* UTI89, demonstrating that curli-producing matrix cells occupied oxygenated outer ridges of rugose colonies, while non-producing washout cells populated hypoxic luminal regions enriched for nitric oxide–related respiration. Oxygen availability and the FNR anaerobic regulator were shown to govern matrix production and spatial organization.

Invited speaker **Jintao Liu** (Tsinghua University, Beijing, China) concluded the session with quantitative approaches that resolve spatial heterogeneity in biofilms. His microfluidic platform generates “pancake-shaped” *E. coli* and *Pseudomonas* biofilms, enabling precise control of growth conditions and high-resolution imaging. These studies revealed that the spatial gradient of glucose led to diffusion-limited growth restricted to the biofilm periphery, and low membrane potential that restricted antibiotic accumulation in the biofilm interior (25). His group also developed RAINBOW-Seq (26), a spatial transcriptomics method which encodes spatial information with growth-dependent fluorescent dyes that label biofilm layer-by-layer as they form, allowing flow-sorting of cells by radial position (Fig. 4B and C). The method revealed sharp distinctions between peripheral growth zones and metabolically specialized interiors, with peripheral cells expressing genes for active growth and secretion of metabolites such as arginine and organic acids, whereas interior cells produced polyamines that modulate peripheral physiology. Liu also discussed how additional gradients of environmental factors further structured biofilm behavior. Using agar block biofilms, he demonstrated that different combinations of glucose and oxygen gradients resulted in different modes of metabolism, leading to non-monotonic relationships between glucose concentration and biofilm growth. He further reported how the metabolic products promoted antibiotic tolerance across spatial domains.

## SESSION 6: MECHANISMS OF BIOFILM DISPERSAL

As important as the formation of biofilms is their dispersal and dissolution to produce free-living microbes. Natural dispersal mechanisms underlie the ability of biofilms to function as recalcitrant reservoirs of re-colonization and disease. Conversely, understanding and gaining control of biofilm dispersal mechanisms may allow novel targeted therapies to disrupt biofilms. This session highlighted the diverse mechanisms and signaling pathways, ranging from c-di-GMP modulation and membrane stress sensing to outer membrane vesicles (OMVs) delivering matrix-degrading enzymes that contribute to biofilm dispersal. Other aspects covered included the efficiency of inducing dispersion using exogenous factors and processes affecting the transition of dispersed cells to the classical planktonic cell phenotype.

Invited speaker **Chris Waters** (Michigan State University, East Lansing, MI, USA) described the c-di-GMP regulatory network that governs the transition of *V. cholerae* between biofilm formation and dispersal. As an environmentally versatile pathogen, *V. cholerae* relies on precise modulation of c-di-GMP through regulators such as VpsR and VpsT, which promote biofilm formation, FlrA, which enhances motility while repressing *vpsR* and *vpsT,* and the phosphodiesterase CdgC, which functions as a key negative-feedback regulator. Competition experiments using bead colonization demonstrated that *cdgC* mutants are less fit than wild type, underscoring the importance of controlled c-di-GMP turnover for dispersal. Waters then described how environmental cues shape c-di-GMP signaling. Within the *Vibrio* seventh pandemic island 2 (VSP-2), he characterized a zinc-regulated phosphodiesterase, ZpdA (27). The *zpdA* gene was found to be repressed by the zinc-responsive regulator Zur, and the ZpdA protein is under direct allosteric negative control by zinc. High zinc concentrations (e.g., chitinous marine substrates) enhanced biofilm formation and inhibited PDE activity, whereas low zinc levels (e.g., seawater) derepressed *zpdA,* promoting planktonic growth. Waters’ lab members are also applying a novel approach called SoRB-TIS (Sorting Random Bar-coded Transposon Insertion Sequencing) to more broadly identify additional c-di-GMP sensors and regulatory components in *V. cholerae*.

One of the factors that contributes to biofilm dispersion in *P. aeruginosa* and multiple other bacteria is the production of OMVs. Extracellular vesicles are produced by all domains of life, yet their roles have been characterized primarily in bacteria grown planktonically. Invited speaker **Jeffrey (Jeff) Schertzer** (Binghamton University, Binghamton, NY, USA) presented an exploration of OMVs as active contributors to *P. aeruginosa* biofilm development and dispersal, focused on a model in which the *Pseudomonas* quinolone signal (PQS) promotes outer-membrane curvature to drive vesiculation. PQS levels rise sharply during biofilm dispersal, with the highest levels in dispersed cells. Mutants in *pqsA*, driving the first step of PQS synthesis, have decreased biofilm OMV production and, consequently, reduced dispersion (28, 29). Analysis of OMV cargo revealed them to be enriched in protease, lipase, and DNase activities, highest in OMVs from dispersed cells, and least in OMVs from planktonic cells. OMVs thus appear to deliver matrix-degrading enzymes that facilitate matrix breakdown and cell release. Schertzer further demonstrated that OMV-mediated transfer extends across species boundaries, and dual-species co-cultures exhibit increased OMV production. This likely results from cross-species induction; closely related organisms respond to PQS by producing OMVs, and OMV formation in *P. aeruginosa* can be stimulated by supernatants from other bacterial species. Collectively, Schertzer’s work positions OMVs as dynamic, enzyme-laden effectors that promote biofilm dispersal and shape microbial community behavior.

Along with the role of OMVs, outer membrane stress can clearly contribute to bacterial dispersal and limit biofilm formation. **Emmy Nguyen** (Laboratory of Andrew Bridges, Carnegie Mellon University, Pittsburgh, PA, USA) described DbfQRS, a membrane-stress–sensing, phosphorelay pathway that regulates dispersal and biofilm formation for *V. cholerae*, controlling genes involved in biofilm matrix synthesis, central metabolism, peptidoglycan synthesis, and other stress responses. Building on earlier work, Nguyen characterized DbfQ, a small periplasmic protein, encoded in the *dbfQRS* operon. Deletion of either *dbfS* or *dbfQ* abolished dispersal, confirming their functional linkage. Biochemical and genetic evidence revealed DbfQ binding to the DbfS histidine kinase, decreasing phosphorylation of the DbfR response regulator and promoting dispersal (30). Outer membrane perturbations, due to LPS defects or membrane-damaging antimicrobials, also activated DbfR phosphorylation. These findings elucidate how outer membrane stress can engage the DbfQSR system and impact the tendency of *V. cholerae* to disperse.

When biofilms of *Staphylococcus epidermidis* are dispersed, they form cell clusters of different sizes and shape distributions, with oblate and larger-sized clusters that often can still avoid immune clearance. **Elizabeth Stewart** (Worcester Polytechnic Institute, Worcester, MA, USA) presented a machine-learning–enabled analysis of *S. epidermidis* to sort cell clusters released from biofilms using confocal laser scanning microscopy coupled with quantitative image analysis. This approach resolved five distinct cluster types defined by shape, diameter, and number of cells per cluster. Among chemical disruptors tested, sodium meta-periodate targeting the polysaccharide intercellular adhesin (PIA) was found to be the most effective in releasing cell clusters from biofilms (31). Stewart emphasized that cell cluster size and morphology are biologically relevant, as oblate and larger aggregates may evade or impair phagocytic clearance.

Although dispersion is often assumed to rapidly restore planktonic physiology, it has been observed that after dispersal from biofilms, bacteria can maintain a distinct non-planktonic phenotype (32). **Manmohit Kalia** (Laboratory of Karin Sauer, Binghamton University, Binghamton, NY, USA) examined the length of time *P. aeruginosa* dispersed cells retain their distinct post-dispersal phenotype, finding that dispersed cells exhibit prolonged differences in growth, signaling, and host interactions. Dispersed cells displayed an extended lag phase, reduced growth rate, altered c-di-GMP levels, and elevated virulence gene expression, all of which required 6–12 hours to revert to planktonic levels. In contrast, immune-evasive behavior persisted for at least 24 hours, indicating a slower reversion of host-interaction traits. Kalia further demonstrated that soluble polysaccharides released during dispersion can induce planktonic cells to adopt dispersed-like phenotypes, including aberrant growth and phagocytosis avoidance. These findings reveal that dispersed cells transition back to a classical planktonic state in a trait-specific, stepwise manner and retain dispersion-associated properties long after leaving the biofilm.

## SESSION 7: SOCIAL AND ASOCIAL INTERACTIONS IN BIOFILMS

Microbial communities exhibit diverse social activities, including coordination of movement, engagement in chemical warfare, genetic exchange, perception of kin, and the evolution of biofilm determinants under changing conditions. This session highlighted many of these different aspects of microbial sociobiology.

Invited speaker **Jo Handelsman** (University of Wisconsin-Madison, Madison, WI, USA) opened the session with an exploration of a model microbial community isolated from the rhizosphere (roots and the surrounding soil that they influence), described as THOR “The Hitchhikers Of the Rhizosphere,” composed of *Pseudomonas koreensis*, *Bacillus cereus*, and *Flavobacterium johnsoniae*. Within this system, *B. cereus* plays a key role as a plant disease suppressor and antibiotic producer, yet nearly 5% of soil *Bacillus* isolates carry microbial “hitchhikers,” including *F. johnsoniae*. Although *F. johnsoniae* does not grow on root exudates alone, it does consume sloughed peptidoglycan and grows in co-culture with *Bacillus* isolates that detoxify inhibitory metabolites. Another hitchhiker, *P. koreensis*, functions as a linchpin member of THOR by producing the koreenceine (aka “THOR’s hammer”), which acts to regulate genes in all community members. A striking feature of this community is the collective movement mediated by *F. johnsoniae*, which moves across surfaces using gliding motility. EPS-enclosed balls or “Zorbs” can encapsulate other bacteria, including *S. aureus* and *P. aeruginosa*. Similar Zorbs form *in vivo*, such as in colonized zebrafish tissues, including those in the brain, suggesting that this collective motility can influence pathogenicity.

Shifting from cooperative movement to competitive interactions, **Ruth Isenberg** (Laboratory of Julia Willett, University of Minnesota, Twin Cities, MN, USA) presented work on streptococcal natural products in dual-species biofilms. Co-cultures of *E. faecalis* with *Streptococcus gordonii* or *S. sanguinis* showed minimal antagonism, whereas *Streptococcus mutans* killed *E. faecalis*. This antagonistic activity extended to other gram-positive bacteria, including other *E. faecalis* strains and *S. aureus*. Genetic analysis in *S. mutans* revealed that the lethal interaction with *E. faecalis* depended on *mubD,* required for production of the cyclic lipopeptide mutanobactin. Deletion of *mubD* abolished antagonism in the model strain; however, natural isolates of *S. mutans* devoid of mutanobactin production antagonized *E. faecalis,* suggesting additional or parallel mechanisms. In some cases, *E. faecalis* populations recovered after prolonged co-culture, and this recovery depended on the secreted protease GelE.

The lysis of cells via bacteriophage infection can induce collective behavior in certain bacteria. **Yangyang Liu** (Laboratory of Andrew Bridges, Carnegie-Mellon University, Pittsburgh, PA, USA) was an Early Career Symposium awardee (Table 4) who described how a lysis-sensing pathway drives collective aggregation in *Vibrio parahaemolyticus*. Exposure to bacteriophage-induced *V. parahaemolyticus* lysate triggered rapid aggregation during recovery, with self-lysate sufficient to initiate this response. A comprehensive transposon mutagenesis library screen hinted that lysis sensing and aggregation depended on production of an iron-chelating siderophore called vibrioferrin. Lysate exposure downregulated vibrioferrin production, whereas vibrioferrin synthesis or receptor mutants failed to aggregate appropriately. The results suggest that vibrioferrin can serve as a signal that enables *Vibrio* populations to detect cell lysis and subsequently aggregate. Horizontal gene transfer is a profound form of social interaction that can impact and, in turn, can be fostered by microbial communities. **James (Jay) Winans (Laboratory of Carey Nadell**, Dartmouth College, Hanover, NH, USA) has investigated how conjugation influences organization and resilience in mixed-species biofilms. Using flow-cell biofilms composed of *E. coli* and *V. cholerae*, Winans employed fluorescent reporters to track single-cell plasmid transfer events in biofilms and observed that conjugative transfer reshaped cell packing over time. Notably, the conjugative pili themselves contributed to biofilm cohesion, creating communities that were markedly more tolerant to phages and antibiotics than plasmid-free populations. This increased resilience to exogenous threats came at the cost of efficient dispersal, such that in dispersal and biofilm recolonization assays, plasmid-free cells could frequently outcompete plasmid-carrying cells globally, even as plasmid carriers produced more robust biofilms in local environments. This highlights how some traits are beneficial in the biofilm setting but costly for the individual free-living cell.

The evolution of biofilm formation can be driven by social interactions among competing sub-populations. The session concluded with Invited speaker **Olaya Rendueles** (Center for Integrative Biology, Toulouse, France), who presented work on determinants of biofilm formation in the opportunistic pathogen *Klebsiella pneumoniae*. A central focus was the capsule, the glycan composition and regulation of which shapes adhesion and biofilm development. Across diverse *K. pneumoniae* strains, the relationship between capsule production and biofilm formation was complex, varying in a strain- and capsule-dependent manner. Experimental evolution in nutrient-rich and nutrient-poor environments—including artificial urine and sputum—suggested negative frequency-dependent selection with initially poor biofilm formation evolving over time to increased amounts of biofilm. In low-nutrient conditions, there was frequent emergence of a novel rdar-like colony morphotype (rough, dry, and red—when grown on media with the dye Congo Red) under negative frequency-dependent selection. These mutants were mediated by mutations in *mrkH* (type III fimbriae regulator) and *nac* (a global nitrogen-responsive regulator). These mutants produced fewer fimbriae but displayed higher fitness in mixed cultures. Similar genotypes (e.g., *mrkH* mutants) were identified among bloodstream isolates and in global genomic databases, indicating their potential clinical relevance.

## SESSION 8: BIOFILMS ON HOST ORGANISMS

Biofilms are clearly of tremendous importance to host interactions, from pathogenesis to commensal interactions. This session highlighted how pathogens ranging from *Pseudomonas* and *Salmonella* to invasive *E. coli* form aggregates or biofilms *in vivo*, with the aggregative growth mode driven by oxygen gradients, immune pressure, nutrient availability, and cell-surface structures such as O-antigen or cellulose. Multiple presentations emphasized how biofilm formation is not a single phenotype but a spatially structured, environmentally responsive strategy that shapes microbial survival, host interactions, disease outcomes, and beneficial interactions.

Invited speaker **Daniel Wozniak** (Ohio State University, Columbus, OH, USA) opened the session with an examination of *P. aeruginosa*–driven skin infection and the interactions between host and pathogen that shape disease progression. Using an intradermal mouse infection model, his group demonstrated that live *P. aeruginosa* induced persistent lesions accompanied by a robust inflammatory response, with bacteria remaining detectable for up to 3 weeks. Host RNA-seq profiling revealed that infected skin rapidly becomes hypoxic and enriched in oxidative stress signatures (33). Neutrophils dominated the early immune infiltrate in lesions, and their NADPH oxidase activity likely drove elevated reactive oxygen species. Antioxidant treatment reduced tissue pathology, underscoring the contribution of oxidative stress to disease severity. Early neutrophil depletion markedly increased bacterial burden, dissemination, and mortality, whereas later depletion had minimal impact, highlighting the importance of the initial neutrophil response. Bacterial transcriptomics from biopsies of early-stage wounds also showed strong induction of an oxidative stress response and alginate biosynthesis genes, while the biosynthesis genes of other adhesive biofilm polysaccharides, *pel* and *psl,* were not differentially expressed. These early transcriptional changes paralleled a suppression of motility and a rise in intracellular c-di-GMP. Wozniak discussed how these features align with established criteria linking *P. aeruginosa* biofilms to infection, including surface adherence, EPS production, spatial confinement, and persistence despite immune pressure, consistent with the transition to a biofilm-like state within the infected skin microenvironment (34).

**Fig 6:**
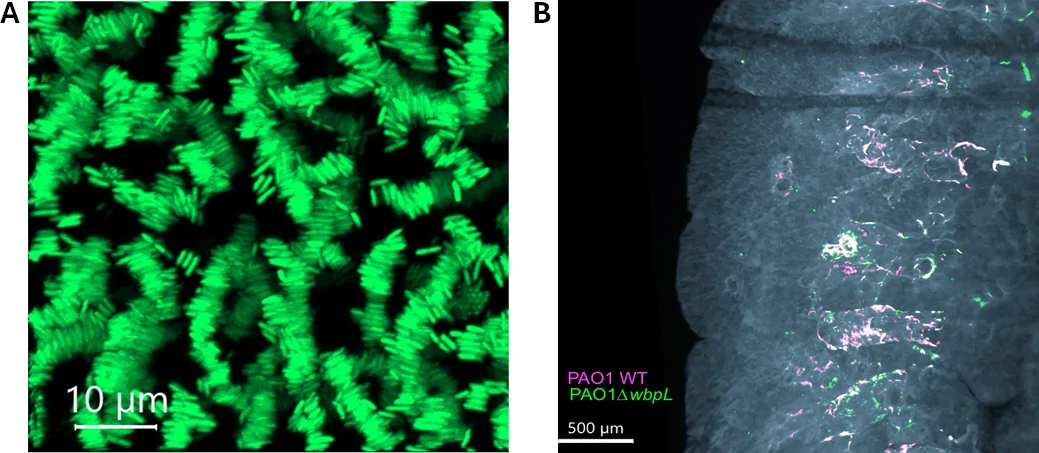
Confocal image showing pancake-shaped stacked *P. aeruginosa* aggregates (**A**) formed *in vitro* and (**B**) mixed aggregates formed by *P. aeruginosa* PAO1 and *wbpL* mutant in mouse airways. Images kindly provided by Sheyda Azimi.

LPS is well established as one of the surface features to which the mammalian immune system responds during bacterial infection. **Sheyda Azimi** (Georgia State University, Atlanta, GA, USA) presented work on how O-antigen (OA) heterogeneity shapes polymicrobial biofilm architecture in chronic infections (35). Chronic wounds and bronchiectasis exhibit persistent spatial patterns of *P. aeruginosa* aggregates, driven in part by cell-surface hydrophobicity (36). Using a synthetic sputum medium (37) in a chamber slide combined with live cell imaging of cells, *P. aeruginosa* formed “pancake-like” stacked aggregates (rather than typical mushrooms, Fig. 6). Azimi showed that loss of OA disrupts the characteristic aggregates that form during infection and interferes with wild-type assembly during co-inoculation with an OA-deficient *wbpL* mutant. *In vivo* imaging using tissue clearing and mRNA HCR-FISH revealed that *wbpL* mutants form more concentrated aggregates within small airways. Despite a competitive fitness equivalent to wild type, this resulted in the formation of larger aggregates by wild-type cells in mouse airways. These findings, supported by recent work in mouse infection models (38), demonstrate that OA diversity profoundly influences aggregate size and spatial organization, possibly contributing to persistence and pathogenicity of chronic infections.

Additional cell surface structures that are well recognized to mediate biofilm formation are curli filaments and cellulose in enteric pathogens such as *E. coli* and *Salmonella* sp. **Hans Steenackers** (KU Leuven, Leuven, Belgium) examined *csgA* (curli-deficient), *bcsA* (cellulose-deficient), and *csgD* regulatory mutants of *Salmonella enterica serovar* Typhimurium in different host environments. Colonization of *Salmonella* was evaluated using a microbial simulator of porcine intestinal mucosal ecosystem referred to as M-SPIME, in which bacteria are inoculated into an *in vitro* ileum vessel composed of mucin beads, intestinal slurry, and pancreatic juice. Using M-SPIME, Steenackers demonstrated that neither curli nor cellulose affected colonization, while the addition of fecal slurry decreased the total bacterial load. In a dynamic multi-compartment M-SPIME system that harbors separate vessels for each intestinal compartment, biofilm-competent strains showed a fitness advantage specifically in the mucus-rich ileum. Shifting to whole-organism models, the biofilm-deficient mutants colonized the nematode host *Caenorhabditis elegans* more efficiently yet did not increase disease burden. In contrast to a mouse model, cellulose-deficient mutants outcompeted wild type and triggered heightened inflammation and microbiome disruption, suggesting that in this model system, cellulose may limit virulence during acute infections.

In some disease scenarios, biofilm formation can be directly targeted by mammalian anti-bacterial factors. Studying inflammatory bowel disease, **David Boone** (Indiana University School of Medicine, Indianapolis, IN, USA) described how the stomach-derived anti-amyloidogenic protein gastrokine-1 (Gkn1) shapes gut microbial ecology and host susceptibility (39, 40). Gkn1 coated luminal bacteria and inhibited *E. coli* curli amyloid fiber formation, suppressing biofilm development but not dramatically affecting viability. Gkn1-deficient mice exhibited impaired clearance of invasive *E. coli* LF82, which forms biofilm-like structures in the colon, and these animals showed heightened vulnerability to T-cell–mediated colitis. Notably, Gkn1 loss also leads to motor deficits and brain α-synuclein aggregation, suggesting a mechanistic link between microbial and host amyloids, gut inflammation, and Parkinson-like pathology.

Moving away from mammalian disease systems, invited speaker **Cara Haney** (University of Pittsburgh, Pittsburgh, PA, USA) closed the session with an exploration of how plant-associated *Pseudomonas* species adapt to and protect the rhizosphere. The plant rhizosphere is enriched in overall microbial abundance but decreased in microbial diversity because plant exudates select for specific microbes. Roots act as the primary site of nutrient uptake, and from that perspective might be viewed similarly to the animal gut for microbiome interactions. Although single, specific microbial strains can be sufficient to confer benefits, including nutrient acquisition and pathogen suppression (41), little is known about the mechanism at the molecular level. Using the model plant *Arabidopsis thaliana* and *Pseudomonas brassicacearum* (Pb), Haney’s lab applied a TnSeq approach to identify bacterial genes required for rhizosphere fitness in both wild-type and immunocompromised plants. The diguanylate cyclase–phosphodiesterase MorA was essential for colonization, with *morA* mutants exhibiting hyper-biofilm formation that triggered plant immune activation and impaired growth. The putrescine acetyltransferase SpuC also emerged as a key determinant, suggesting that certain polyamines act as *in vivo* signals promoting Pb biofilms on roots (42). Haney then compared genetic requirements for biofilm formation *in vitro* and *in planta,* finding that some biofilm-deficient mutants of this beneficial pseudomonad failed to protect plants from pathogens, but that biofilm formation as defined *in vitro* only partially correlated with beneficial plant–microbe interactions (43).

## SESSION 9: ANTIMICROBIAL THERAPIES AND BIOFILMS

The use of antibiotics and related therapies to battle biofilms has been a major area of study for many different labs, with clinical relevance and great potential for improving treatment outcomes. Session 9 focused on emerging strategies to understand, prevent, and treat biofilm-associated infections, highlighting innovations ranging from targeted drug delivery platforms to microbial ecology, phage–biofilm interactions, and evolutionary dynamics. Collectively, the talks emphasized that biofilm infections persist not simply because bacteria are resistant, but because biofilms create complex physical and physiological barriers that protect metabolically diverse bacterial populations. The speakers presented complementary approaches, spanning from targeted drug delivery to microbial ecology, phage–biofilm interactions, and evolutionary dynamics, that collectively point toward new therapeutic opportunities.

Invited speaker **Rikke Louise Meyer** (Aarhus University, Aarhus, Denmark) opened the session with a comprehensive overview of advanced drug-delivery platforms designed to overcome biofilm antibiotic tolerance, highlighting the extreme drug concentrations required due to poor penetration, stress responses, and persister cells. Her work leverages bacterial enzymes for localized drug release, offering selective killing with reduced toxicity and demonstrating strong translational potential for targeting persistent biofilm states. Meyer outlined her group’s progression from mesoporous nanoparticles (44) and aptamer-targeted liposomes that enable localized antibiotic release (45) to antibody–drug and glycopeptide-directed cytotoxic conjugates to improve eradication of persister cells. Mitomycin-C, a DNA crosslinker used in oncology, proved highly effective against both active and dormant biofilm bacteria while generating little resistance due to its protein-independent mechanism. To reduce toxicity, an antibody–mitomycin conjugate with a cleavable disulfide linker was engineered, enabling bacterial enzyme-triggered release in a mouse model of implant-associated bone infection, with the conjugate outperforming vancomycin (46, 47). To overcome the cost and stability constraints of antibodies, her group repurposed glycopeptide and lipoglycopeptide scaffolds to create modular platforms. For example, vancomycin–mitomycin conjugates bound gram-positive bacteria and killed planktonic and biofilm-embedded cells but showed limited tissue penetration. Lipoglycopeptide dalbavancin–mitomycin conjugates displayed broader activity, including unexpected effects on select gram-negative species, and supported delivery of additional drug classes. Using this platform, a nucleoside analog inactive against persisters became greater than 250-fold more potent while retaining low toxicity. Meyer concluded that glycopeptide- and lipoglycopeptide-directed conjugates represent a powerful and modular strategy for selectively delivering potent drugs to biofilm infections, with strong translational potential for addressing longstanding treatment failures.

**Ankur Sood** (Laboratory of Rosana Ferreira, University of Kansas, Lawrence, KS, USA) shifted the focus from synthetic drug delivery systems to natural biotherapeutics produced by the skin microbiome. Sood’s work centers on the protective role of *S. epidermidis*, a dominant skin commensal that suppresses *S. aureus* virulence without inhibiting its growth. Sood identified pyroglutamic acid from *S. epidermidis* as a commensal-derived molecule that suppresses *S. aureus* biofilm formation, adhesion, and invasion without inhibiting growth, illustrating how host-associated microbial interactions can be mined for antivirulence therapeutics. The work offers a compelling example of how commensal-derived small molecules can be utilized for new therapeutic approaches and underscores the importance of understanding microbial interactions in the host environment. Bringing a biophysical perspective, **Robert Edmiston** (Laboratories of Jennifer Curtis and Steve Diggle, Georgia Institute of Technology, Atlanta, GA, USA) examined how bacteriophage interactions restructured *P. aeruginosa* biofilm populations into highly ordered “stacked aggregates” (Fig. 6A), in stark contrast to the loose, disorganized structures typically formed without phage pressure. These ordered structures arose from alginate overproduction in phage-resistant mutants and conferred enhanced tolerance, demonstrating how evolutionary responses to phage reshape biofilm architecture. This theme was continued by **Sophia Mason** (Laboratory of Matthew Wolfgang, University, North Carolina, Chapel Hill, NC, USA) by showing that airway mucus in people with cystic fibrosis selects for low-biofilm, high-tolerance *P. aeruginosa* phenotypes, providing insight into persistent infections in cystic fibrosis: mucus-rich environments favored low surface-attached biofilm formation, promoting tolerance rather than classical resistance, enabling long-term survival despite aggressive antibiotic therapy. In contrast, populations that evolved without mucus became better biofilm formers, but they were less tolerant. Importantly, tolerance increases occurred without MIC changes, confirming that the effect was true tolerance, not resistance.

Invited speaker **Vaughn Cooper** (University of Pittsburgh, Pittsburgh, PA, USA) concluded the session with an integrative analysis of how biofilm-specific evolutionary dynamics drive antimicrobial resistance and tolerance. Cooper’s foundational work with *Burkholderia* revealed that biofilms diversify extremely rapidly and reproducibly, often through single mutations in key regulatory loci such as *rpfR* and members of the *wsp* pathway (48, 49). Using a plastic-bead model that enforces daily cycles of attachment, dispersal, and reattachment, his group evolved populations under escalating ciprofloxacin treatment. Cooper went on to show that under antibiotic pressure, biofilms by *Acinetobacter baumannii* maintained high genetic diversity, with mutations centering on repeated mutations in regulators of the Ade efflux pump system, especially *adeL* (50). These mutations enhanced both efflux and adhesion simultaneously, showing how single regulatory “knobs” adjust multiple phenotypes at once. Intriguingly, some biofilm-adaptive mutations conferred collateral sensitivity to β-lactams, and β-lactam exposure could even drive *precise reversion* of earlier SNPs, demonstrating that evolutionary pathways are reversible under the right selection pressures (51). In contrast, planktonic populations underwent classic selective sweeps dominated by mutations in DNA gyrase. *In vivo* studies further revealed that immune deficiency accelerates pathogen expansion and promotes tolerance phenotypes before classical resistance mutations arise, suggesting that immune deficiency expands the evolutionary landscape accessible to pathogens and may promote tolerance phenotypes (52). Consistent with the *in vitro* studies, sequencing from immunodeficient mice again identified repeated *adeL* mutations, highlighting conserved pathways that shape biofilm evolution across environments. Cooper concluded by emphasizing that these deeply conserved evolutionary levers represent promising targets for therapeutic intervention and highlighted the educational power of real-time evolution experiments that consistently recapitulate biofilm-adaptive mutations.

## SESSION 10: BIOFILM CONTROL AND ENGINEERING

The control of biofilms in both positive and negative ways is an emerging area of research, with the application of multiple biological and engineering approaches. This session highlighted examples of each class of targeted control of biofilms, with the two invited speakers focusing on each end of this spectrum. **Tammy Kielian** (University of Nebraska Medical Center, Omaha, NE, USA) described work in combating *S. aureus* prosthetic joint infections (PJIs), developing strategies to interfere with the reprogramming of the innate immune response involving the granulocytic myeloid-derived suppressor cells (G-MDSC) class of neutrophils during these PJIs, in which the biofilms cause suppression of a robust immune response (53). Strikingly, this immune suppression involves the transfer of mitochondria from macrophages to G-MDSC cells via tunneling nanotubes, which enhances the oxidative activity in these immune cells (54). Strategies to antagonize these immunosuppressive processes may allow improved clearance of these infections. The second invited speaker for this session, **Anita Shukla** (Brown University, Providence, RI, USA) described a much more engineering-based approach to combat antimicrobial resistance in biofilms, including antibiofilm nanoparticles, surface coatings, and hydrogel-based delivery systems (55). These approaches may be able to augment or even replace antibiotic treatments for biofilm infections. In her talk, Shukla highlighted nanoparticles that could deliver antibiotic payloads into biofilms in matrices comprised of biodegradable materials such as gelatin, chitosan, and hyaluronic acid. Additionally, Shukla discussed engineered hydrogel wound dressings that respond to β-lactamase production by cleavage of the hydrogel and subsequent release of “stored cargo” in the form of an antimicrobial agent. Applications of the nanoparticles and engineered hydrogels in murine wound infections showed fast killing of pathogens and no reappearance of infection. Overall, Shukla’s talk emphasized the capability of engineered materials in drug delivery, suppressing antimicrobial resistance and improving treatment outcomes.

The session covered a wide range of other relevant topics and bacterial systems. In another example of novel engineering in the service of biofilm research, **Cláudia N. H. Marques** (Binghamton University, Binghamton, NY, USA) explained work modeling the environment of the small intestine with a small intestine-on-a-chip (SIOC) system (56). This SIOC “chip” is a microfluidic device that uses a porous membrane to create apical and basolateral layers that are seeded with intestinal cells that establish tissue-like structures under a flow regime. This device was inoculated with a synthetic intestinal microbiota comprised of a fixed inoculum of *E. faecalis, Bifidobacterium bifidum*, *Lacticaseibacillus rhamnosus*, and *Streptococcus salivarius*, expressing different fluorescent labels. In a microscopically accessible format, the SIOC accurately models many of the attributes of the small intestine, including microbial dynamics, their impact on cellular activities, and the resulting cellular structures. **Ana Barbosa** (Laboratory of Professor Nuno F. Azevedo, University of Porto, Porto, Portugal) presented intriguing work on the spatial gene expression profiling of *Legionella pneumophila* biofilms using peptide-nucleic acid fluorescence *in situ* hybridization (PNA-FISH) with single-cell resolution. For biofilms formed in a flowing system, the PNA-FISH approach revealed the expression patterns for key genes associated with both the transmissive and replicative phases of *L. pneumophila*. Given the recognized problem of *L. pneumophila* in water distribution systems, this analysis in a microscopically accessible format and flowing conditions is especially relevant. In contrast to saturated biofilms, **Christopher Jones** (Montana State University, Bozeman, MT, USA) presented the work from his lab on dried biofilms. Dehydrated biofilms can retain viability for months and can be the source of hospital-acquired infections. By examining multiple biofilm-forming bacteria under a variety of drying regimens, Jones and colleagues found that tolerance to drying varied between different taxa and was impacted by the process by which they were dried. Improved understanding of these properties will provide better strategies for limiting infection and contamination from medical contexts to food preservation to industrial applications.

## SESSION 11: HISTORY AND NEW APPROACHES

A historic perspective on biofilms kicked off this session from invited speaker **Hans-Curt Flemming** (Institute of Oceanology, Qingdao, China), focusing on the development of our current understanding of the complex biofilm exopolymeric matrix (Box 5). However, the remaining session moved forward to highlight new approaches for examining spatial structure and physiological heterogeneity of biofilms and droplet-based microfluidic systems. One of the two invited speakers in this session, bioengineer Dr. **Connie Chang** (Mayo Clinic, Rochester, MN, USA) presented her lab’s work on drop-based microfluidic capture of biofilm bacteria (57). By encapsulating cells within picoliter droplets and quantifying fluorescence as a proxy for growth, her group can determine antibiotic susceptibility with remarkable speed. These rapid MIC measurements closely matched traditional assays, with discrepancies arising mainly when antibiotics alter cell morphology. Chang highlighted the translational potential of this technology for tailoring antimicrobial therapies, particularly for infections involving biofilm-embedded or slow-growing bacteria that evade standard diagnostics. This technology holds great promise for increased efficiency in determining antibiotic sensitivities, including for biofilm bacteria, and for rapidly customizing antibiotic therapies to optimize treatments for patients.

Box 5. **The History of Slime—Hans-Curt Flemming**

Invited speaker Professor Emeritus Hans-Curt Flemming (Institute of Oceanology, Qingdao, China) presented a broad perspective on the biofilm matrix and the long development of our current understanding of this complex exopolymeric network (58). His comprehensive presentation described the complex structure and properties of the biofilm matrix, which specific factors determine these properties, and how this material is much more than “slime” (see also Fig. 3). It is now clear that multiple different hydrated biopolymers can comprise the matrix, prominently including exopolysaccharides, proteins, lipids, and nucleic acids, as well as outer membrane vesicles. Additionally, metal ion composition can have a profound effect on the material properties of the matrix. In addition to determining biofilm structure and providing connections between cells, the matrix can also retain enzymes, providing an extracellular digestion system. Furthermore, it hosts non-enzymatic activities, serving to coordinate and partition the complex physiologies manifested by biofilms. Flemming articulated a view where environmental factors and the endogenous genetic circuitry combine to regulate production of the matrix, and thus determine the properties of the overall biofilm, granting them their enormous diversity. The matrix endows biofilms with emergent properties that are only possible in matrix-enclosed aggregation, allowing for multi-tasking. He also emphasized that most of our current information is extrapolated from a few model organisms and that environmental biofilms and their matrices are far less well understood and deserve more attention.

A different droplet-based, microfluidic platform was also reported by **Caroline Miller** (Laboratory of Sophie Darch, University of South Florida, Tampa, FL, USA) to generate *P. aeruginosa* aggregates of defined size, enabling transcriptional profiling of droplet colonies in response to antibiotic treatments. Her studies suggested that spatial heterogeneity within aggregates can contribute to tolerance to colistin, one of the last-line antibiotics for treating multidrug-resistant infections. Transcriptional profiling at single-cell resolution emerged as a recurrent theme throughout the conference. In this session, **Nasibeh Arabameri** (Laboratory of Boo Shan Tseng, University of Nevada, Las Vegas, NV, USA) presented work that earned one of two Early Career Symposium awards (Table 4), highlighting her single-cell analysis of *P. aeruginosa* biofilms. Using a probe-based hybridization method, she identified multiple distinct subpopulations within *in vitro* biofilms based on transcriptional signatures. Several clusters displayed notable features, including one enriched for R2/F2 pyocin production. **Matthew Fields** (Montana State University and Center for Biofilm Engineering, Bozeman, MT, USA) shifted the session toward environmental systems, presenting methods for examining surface-associated microbial communities in terrestrial subsurface and groundwater environments (59). Using innovative porous sampling devices deployed in high-stress sites, his group visualized the spatial distribution, density, and activity of *ex situ* bacterial populations. Contaminated environments showed markedly higher bacterial abundance and metabolic activity but reduced species richness, dominated by acid-tolerant *Rhodanobacter* species commonly isolated from polluted sites (60). These approaches provide new insight into microbial community structure and function in challenging natural environments.

## KEYNOTE PRESENTATION: NICOLE DUBILIER

**Fig 7:**
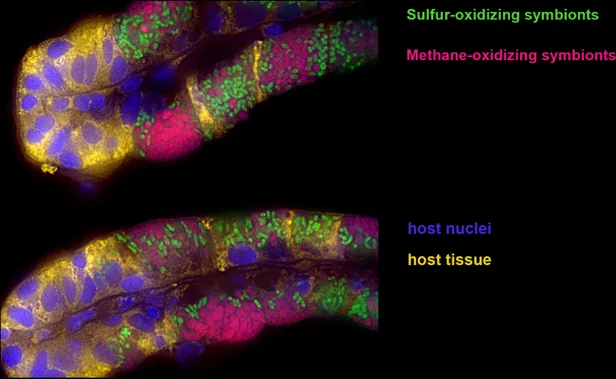
FISH image showing that sulfur- and methane-oxidizing symbionts co-occur in mussels from vents on the Mid-Atlantic Ridge. Image kindly provided by A. Pernthaler and N. Dubilier.

Keynote speaker and marine microbiologist **Nicole Dubilier** (Max Planck Institute for Marine Microbiology, Bremen, Germany) provided a fascinating presentation on the association between symbiotic bacteria and deep-sea invertebrates from hydrothermal vents and cold seeps. Focusing primarily on the symbionts of deep-sea mussels (bathymodiolines), Dubilier described how these animals host dense populations of chemosynthetic bacteria within specialized gill tissues, where the bacteria oxidize sulfide, methane, and hydrogen to generate the primary energy and carbon sources for their hosts. The dense bacterial populations colonizing these tissues form highly organized, host-associated biofilms with specialized metabolic programs that support the symbiosis (61). High-resolution microscopy revealed that the symbionts reside not within host cells, as previously assumed, but in an “inside-out” epithelial pouch accessible to seawater (Fig. 7). These communities consist primarily of multiple strains of Gammaproteobacteria whose genomes display striking plasticity, characterized by abundant insertion sequences and extensive rearrangements. The interaction appears largely bacteria-driven, with minimal contribution from mussel genetics. Candidate bacterial proteins enabling symbiosis are suspected to include secretion systems, such as the Type IV systems capable of delivering effectors into the host cells. A highly abundant beta-propeller protein, likely secreted via a Type I pathway, is a candidate for a direct effector in mediating the symbiotic interactions. Overall, the keynote highlighted the intricate cellular, genomic, and biochemical foundations of deep-sea symbioses and underscored how biofilm-related traits such as structured colonization, metabolic integration, and coordinated community behavior underpin the success of these partnerships.

## SUMMARY AND PERSPECTIVES

This conference was the 10th in an impressive series of ASM meetings focused on biofilms over a span of nearly 30 years. Several of these first meetings were organized by Bill Costerton in the late 1990s and early 2000s, and they started relatively small, but over time, they have grown in the breadth of biofilm research they present, and the range of different scientists that contribute to them. The 10th ASM Conference on Biofilms was a remarkable meeting which highlighted impressive new insights into biofilms using new technologies and approaches, including microfluidics, single-cell genomics, and illuminating new high-resolution microscopic approaches. Model systems continue to provide new insights, but the embrace of more natural contexts for biofilms and their impact on the environment is clearly an emerging area, as is the incorporation of multispecies biofilm models. The training and formative experiences provided for early-career scientists have developed into a major focus for the conference, helping to catalyze the exchange of the latest findings in biofilm research. Despite notable challenges prior to the conference regarding VISAs and other travel authorizations, conferees from outside the United States made a robust showing and, as in past meetings, were crucial to the success of the conference. International participation has always been an important aspect of the ASM Conference on Biofilms, and we hope to continue to see a high level of international contributions at future meetings.

Reflective of this sentiment, the conference concluded with the announcement that ASM intends to support an 11th ASM Conference on Biofilms scheduled for 2028, and co-chaired by Karin Sauer and Kendra Rumbaugh.
